# Visual Target-Driven Robot Crowd Navigation with Limited FOV Using Self-Attention Enhanced Deep Reinforcement Learning

**DOI:** 10.3390/s25030639

**Published:** 2025-01-22

**Authors:** Yinbei Li, Qingyang Lyu, Jiaqiang Yang, Yasir Salam, Baixiang Wang

**Affiliations:** 1 College of Electrical Engineering, Zhejiang University, Hangzhou 310027, China; li_yinbei@zju.edu.cn (Y.L.); yasir.salam@zju.edu.cn (Y.S.); 22210171@zju.edu.cn (B.W.); 2 College of Design and Engineering, National University of Singapore, Singapore 119077, Singapore; e1499227@u.nus.edu

**Keywords:** crowd navigation, deep reinforcement learning, mapless navigation, twin-delayed deep deterministic policy gradient (TD3)

## Abstract

Navigating crowded environments poses significant challenges for mobile robots, particularly as traditional Simultaneous Localization and Mapping (SLAM)-based methods often struggle with dynamic and unpredictable settings. This paper proposes a visual target-driven navigation method using self-attention enhanced deep reinforcement learning (DRL) to overcome these limitations. The navigation policy is developed based on the Twin-Delayed Deep Deterministic Policy Gradient (TD3) algorithm, enabling efficient obstacle avoidance and target pursuit. We utilize a single RGB-D camera with a limited field of view (FOV) for target detection and surrounding sensing, where environmental features are extracted from depth data via a convolutional neural network (CNN). A self-attention network (SAN) is employed to compensate for the limited FOV, enhancing the robot’s capability of searching for the target when it is temporarily lost. Experimental results show that our method achieves a higher success rate and shorter average target-reaching time in dynamic environments, while offering hardware simplicity, cost-effectiveness, and ease of deployment in real-world applications.

## 1. Introduction

**Figure 1:**
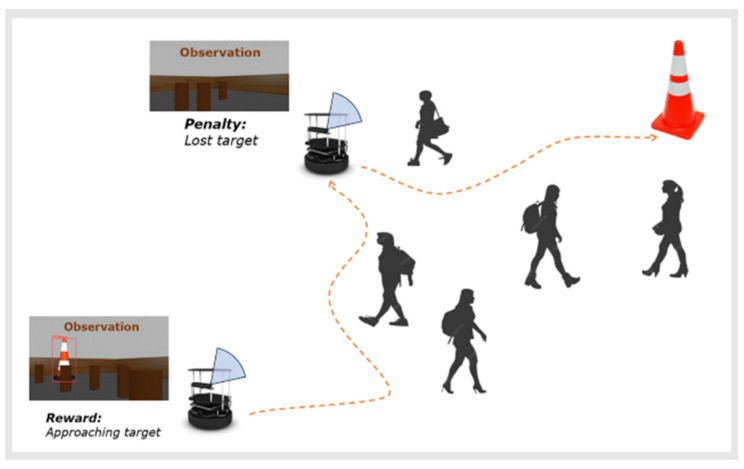
A scenario of a mobile robot navigating in a crowded environment toward a visual target.

Effective navigation in crowded and dynamic environments, as illustrated in Figure 1, is critical for a wide range of real-world robotic applications, such as autonomous delivery robots maneuvering through busy urban streets, service robots operating in congested shopping malls, and search-and-rescue robots deployed in disaster-stricken areas. Traditional navigation methods, such as those based on Simultaneous Localization and Mapping (SLAM), which rely on pre-mapping of environments, often underperform in these complex settings. The main limitation of SLAM-based approaches is their dependence on static maps and the assumption that the environment remains relatively unchanging [1,2], resulting in frequent localization errors and suboptimal navigation paths in dynamic environments.

In response to this challenge, deep reinforcement learning (DRL) has emerged as a promising solution. DRL’s ability to learn directly from interactions with the environment enables it to adapt to a wide variety of scenarios, making it particularly effective in unknown and dynamic environments [3,4,5]. Unlike conventional methods, DRL-based approaches do not require a predefined map, instead modeling navigation tasks as Partially Observable Markov Decision Processes (POMDPs) [6,7], an extension of Markov Decision Processes (MDPs). A POMDP is defined by the tuple:

(1) $$M=\left(S,\;A,\;T,\;R,\;Ω,\;O,\;\Upsilon \right)$$

where S represents the state space, A denotes the action space, T is the transition probability, R is the reward function, Ω is the observation space, O is a set of conditional observation probabilities, and Υ is the discount factor [8,9,10]. DRL techniques, including specific algorithms such as Deep Q-Networks (DQNs) and Proximal Policy Optimization (PPO), as well as broader frameworks like Actor-Critic methods, have demonstrated significant potential in enabling robots to navigate dynamic environments without relying on prebuilt maps. These approaches effectively handle the complex, sequential decision-making processes inherent in such tasks [11,12].

However, existing DRL-based mapless navigation solutions are often constrained by their reliance on complex feature extraction processes and sensor fusion strategies, which typically require systems with a wide field of view (FOV), as shown in [13,14,15,16]. While other methods are effective in relatively static environments as shown in [17,18,19], they struggle in highly dynamic settings characterized by partial observability. In such environments, robots receive incomplete observations, which increases decision uncertainty. Moreover, traditional MDP-based approaches face significant challenges in handling large or continuous state and action spaces, often requiring function approximation techniques that can complicate model convergence and degrade real-time performance. The heavy reliance on handcrafted feature extraction methods exacerbates these issues, reducing generalization capabilities of the learned models.

Navigating in environments with limited FOV introduces additional complexities. The restricted observational capacity hampers a robot’s ability to accurately characterize its state, thereby reducing situational awareness. Furthermore, high-dimensional state representations derived from multimodal sensing data pose substantial difficulties for conventional DRL algorithms, particularly when generating continuous action spaces. This can lead to overfitting, limiting robot’s adaptability to unseen scenarios.

To address these challenges, this paper proposes a DRL-based method specifically for visual target-driven navigation in crowded environments with limited FOV. Our approach is grounded in key principles aimed at enhancing generalization and practical applicability. First, we minimize hardware requirements by employing a single RGB-D camera with limited FOV, thereby reducing dependency on complex localization techniques, and enhancing deployment versatility across a broad spectrum of robotic platforms. This minimalist sensor setup not only reduces costs but also simplifies the overall system architecture, making it more robust and accessible for various applications.

Second, we mitigate the risk of overfitting by incorporating dynamic obstacles into the training process, ensuring that the learned strategies are robust and adaptable to changing conditions in real-world scenarios. By introducing a randomly positioned visual target during training, we prevent the model from becoming overly dependent on specific environmental configurations, thereby enhancing the generalization capabilities.

Furthermore, to overcome the challenge associated with target tracking within a limited FOV, our method integrates a self-attention network (SAN). The SAN infers positional information of the lost target based on past observations, enabling the robot to effectively search for the target.

At the core of our approach is the Twin-Delayed Deep Deterministic Policy Gradient (TD3) algorithm, known for its effectiveness in handling continuous action spaces, to enable efficient navigation in crowded environments. The TD3 algorithm addresses the challenges associated with traditional DRL approaches by introducing several innovations, including twin Q-networks and delayed policy updates, which improve stability and performance in complex environments.

In summary, this paper addresses the gaps in the current literature on DRL-based navigation in crowded environments by proposing a practical solution that reduces both observational and computational demands while maximizing adaptability. Our contributions are threefold:

- We propose a novel DRL-based architecture for mapless navigation in unseen and dynamic environments, which relies exclusively on a visual target without requiring any environment modeling or prior mapping.
- We propose a SAN-based feature extractor to enhance the robot’s ability to search for and track targets in environments with dynamic obstacles and randomly positioned targets.
- Experimental results have demonstrated the superior performance of the proposed architecture in navigating in crowded and dynamic environments, even with a limited FOV.

The remainder of this paper is organized as follows: Section 2 provides a review of related work on DRL-based navigation, with emphasis on visual target-driven approaches. Section 3 details the system architecture of the proposed method. In Section 4, we present the navigation policy representation. Section 5 introduces the training algorithms used for the proposed model. Section 6 presents the experimental results and compares our approach with existing methods. Section 7 discusses the experiment results. Finally, Section 8 concludes the paper and discusses directions for future work.

## 2. Related Work

Current DRL-based navigation systems can be broadly classified into three categories based on the environmental context in which robots operate, namely, map-based navigation, mapless navigation in static environments, and mapless navigation in dynamic environments.

### 2.1. Map-Based Navigation

In environments where a prebuilt map is available, researchers have explored integrating DRL with conventional SLAM to enhance obstacle avoidance capabilities. For instance, Chen et al. [20] proposed a navigation system that combines a DQN-based planner with SLAM to improve navigation performance in dynamic environments by utilizing a prebuilt costmap. Similarly, Shunyi Yao et al. [21] developed a DRL-based local planner for maneuvering through crowded pedestrian areas using a prebuilt map. Although these applications demonstrate improved obstacle avoidance, their reliance on static prebuilt maps limits their adaptability to dynamic environments, reducing their generalization to unseen or evolving scenarios.

### 2.2. Mapless Navigation in Static Environment

In relatively static environments, DRL-based systems have been developed for target-driven navigation without the need for prebuilt maps. Kulhánek et al. [22] demonstrated the efficacy of visual navigation in a static living room environment, using a LSTM network to leverage past actions and rewards to track targets. They proposed the A2C with Auxiliary Tasks for Visual Navigation (A2CAT-VN) framework, optimized for static indoor environments. Hsu et al. [23] proposed a vision-based DRL approach for robots to navigate a large-scale static environment by combining the current state image and last action. Zhu et al. [24] proposed a Deep Siamese Actor-Critic Network, which employs a dual-stream network to embed current observations and target images into a shared space for target-driven navigation, achieving successful navigation in indoor environments without a prebuilt map. Kulhánek et al. [25] further integrated the Parallel Advantage Actor-Critic (PPAC) algorithm with LSTM for indoor navigation without dynamic obstacles, while Wu et al. [26] incorporated an information-theoretic regularization into an A3C framework to enable mapless navigation toward novel targets. Despite these successes, most existing DRL-based visual navigation systems are constrained by their application to relatively static environments. They often rely on complex network architectures and deep feature extraction, which are insufficient to handle navigation in highly dynamic and crowded settings.

### 2.3. Mapless Navigation in Dynamic Environment

Mapless navigation in highly dynamic and crowded environments poses distinct challenges. Researchers have made progress in achieving navigation in dynamic settings without relying on prebuilt maps, though their solutions often depend heavily on sensor fusion and feature extraction techniques. For instance, Anas et al. [13] employed odometry and 2D laser data to develop a collision probability concept, while Shi et al. [14] introduced the notions of Traversability, VO Feasibility, and Survivability to guide navigation. Sun et al. [15] proposed a spatial feature encoder incorporating risk-aware and attention-based feature extraction strategies. Despite these advancements, the heavy reliance on handcrafted features and sophisticated sensor setups, such as combinations of lidar and depth cameras [14], complicates real-world deployment and increases the risk of overfitting to specific environments. Furthermore, most current approaches are tailored to predefined target positions, which can cause overfitting and limit their generalization to unseen scenarios.

In contrast to existing approaches, our method presents several key advantages. First, unlike map-based techniques, our solution enables real-time navigation without requiring prior mapping, making it both more flexible and scalable in unknown scenarios. Second, while most DRL-based methods using visual input are optimized for static environments, our system is specifically designed to operate in dynamic settings. Furthermore, compared to other DRL approaches in dynamic environments, which often rely on complex sensor fusion, we achieve efficient navigation using only a single RGB-D camera. Finally, our approach handles randomly positioned visual targets and reduces the need for handcrafted feature extraction, enhancing adaptability in real-world applications.

## 3. System Architecture

### 3.1. Overall System Design

**Figure 2:**
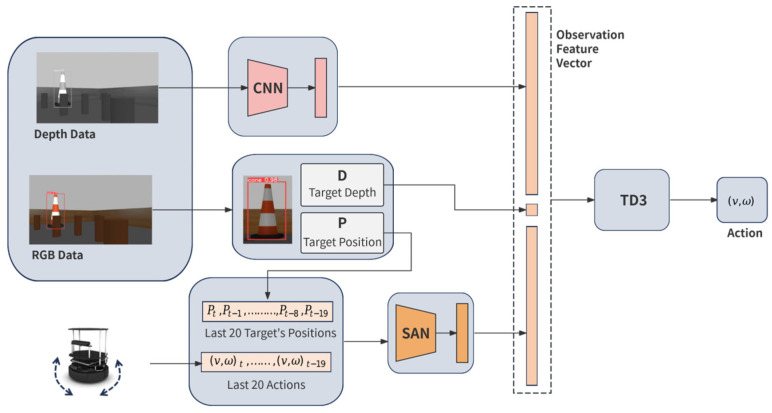
System architecture of the proposed visual target-driven robot navigation.

The architecture of the proposed DRL-based robot navigation approach, as shown in Figure 2, integrates several components, including depth data processing via a convolutional neural network (CNN), YOLOv5-based target detection, a SAN for temporal feature extraction, and TD3 algorithm for policy learning. Collectively, these components form a robust framework for visual target-driven robot navigation in crowded environments.

### 3.2. Depth and Visual Data Processing

**Figure 3:**
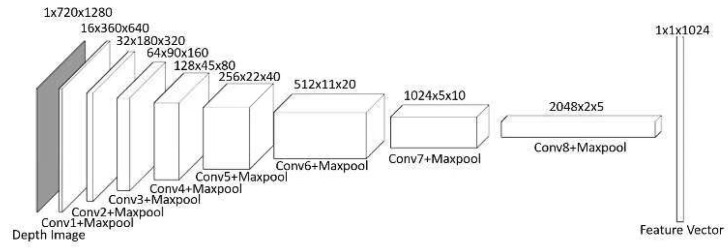
Convolution neural network (CNN) to extract features from depth data.

The system receives input from an onboard RGB-D camera with 70-degree horizontal FOV, a compact setup compared to systems in many existing works assuming a 360-degree FOV [27]. The depth data (1280 × 720 pixels), representing the 3D structure of the environment, are processed through an eight-layer CNN, as shown in Figure 3. Each layer uses 3 × 3 kernels with 2 × 2 max-pooling to down-sample the data, reducing them to a feature representation (2048 × 2 × 5), which is further connected to a fully connected layer and then flattened to feature vector with dimension of 1024 for integration into the decision-making pipeline. The choice of the feature vector’s dimension is guided by a trade-off among TD3 training convergence, the adequacy of environmental representation, and the real-time performance during inference.

In parallel, YOLOv5 is employed to perform real-time target detection using RGB data capture by the same RGB-D camera. YOLOv5 predicts bounding boxes and class probabilities in a single pass, allowing continuous target tracking as the robot navigates. Once the target’s bounding box is detected, the depth value at its center is retrieved from the depth map, enabling the system to estimate the target’s distance and enhance situational awareness during navigation.

### 3.3. Self-Attention Network for Temporal Feature Processing

One key challenge posed by the robot’s limited 70-degree FOV is its restricted ability to perceive the entire environment, making it harder to track the target which moves outside its visual range. To mitigate this, the SAN is employed to compensate for the limited FOV by extracting temporal and spatial dependencies from past observations. This approach is inspired by the SAN’s demonstrated effectiveness in capturing complex dependencies in previous research [28,29]. By learning patterns and relationships between actions and their outcomes over time, the SAN enables the robot to maintain situational awareness beyond its current FOV.

**Figure 4:**
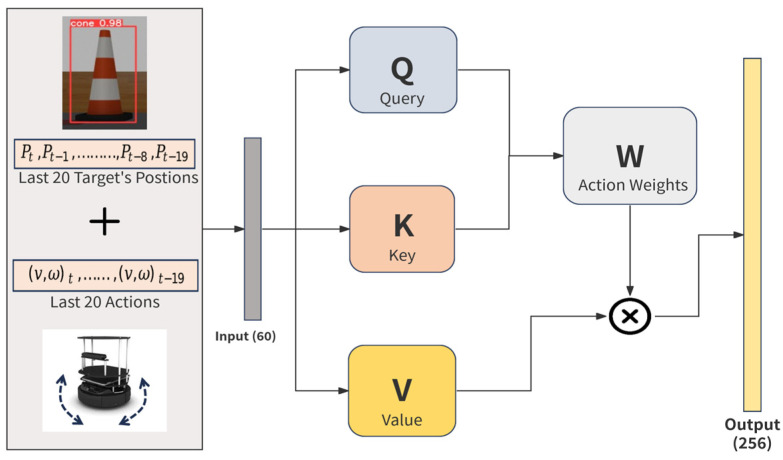
Self-attention network to process sequences of past target positions and actions.

In this study, the SAN processes sequences of 20 past target positions and 20 corresponding actions, producing a 128-dimensional feature vector, as illustrated in Figure 4. This dimension is selected to strike a balance between computational efficiency and the capacity to capture sufficient temporal patterns for effective decision-making. This configuration enables the robot to “reconstruct” a broader understanding of the environment, enabling it to track the target’s location even when it temporarily falls outside the camera’s view. By leveraging these temporal patterns, the SAN improves decision-making in target pursuit in dynamic environments.

The effectiveness of the SAN in our approach lies in its ability to dynamically prioritize relevant spatial and temporal features, allowing the robot to respond when the target is lost from its current view. The self-attention mechanism works by allowing the model to assign varying levels of importance to different parts of its past observations, such as past target locations and actions. This means that when key information, like the target’s location, is missing from the current FOV, the model can attend to previous observations that still hold useful data about the target’s movement patterns. By computing relationships between queries, keys, and values, the robot can better predict where the target is likely to be, even when direct visual input is unavailable. This dynamic allocation of attention enables the robot to maintain robust situational awareness and make more informed navigation decisions, even in challenging environments.

Mathematically, this attention mechanism is represented by the following formula:

(2) $$Attention=Softmax(\frac{QK^{T}}{\sqrt{d_{K}}})V$$

where the robot’s input sequence (i.e., past target positions and actions) is transformed into three distinct matrices—Query (Q), Key (K), and Value (V). Queries assess relevance, Keys represent the elements being attended to, and Values provide the actual data being aggregated. This allows the robot to focus on the most critical parts of its environment and adjust its behavior accordingly, despite the limited FOV.

### 3.4. Twin-Delayed Deep Determinstic Policy Gradient (TD3)

Building on the feature extraction capabilities of the CNN and SAN, TD3 is employed as the core policy learning algorithm to manage continuous action spaces in the crowd navigation task. TD3 enhances stability by using twin critics to reduce Q-value overestimation and delayed policy updates to improve training efficiency [30]. Its strength in continuous action optimization makes it effective for real-time obstacle avoidance in dynamic environments, as demonstrated by its integration with methods like the Dynamic Window Approach (DWA) for LiDAR-based navigation [31] and Long Short-Term Memory (LSTM) networks for path-following in autonomous systems [32]. In dynamic environments with unpredictable obstacle movement, TD3 is advantageous in managing continuous action space for obstacle avoidance. Its twin critics compute conservative value estimates, addressing overestimation bias, which is particularly critical in environments where rapid changes occur. The TD3 loss function is defined as follows:

(3) $$L\left(\theta_{1},\theta_{2}\right)=\frac{1}{N}\;\overset{N}{\underset{j=i}{\sum }}{\left(Q_{\theta_{1}}\left(s_{j},a_{j}\right)-y_{j}\right)}^{2}+\frac{1}{N}\;\overset{N}{\underset{j=i}{\sum }}{\left(Q_{\theta_{2}}\left(s_{j},a_{j}\right)-y_{j}\right)}^{2}$$

where $Q_{\theta_{i}}$ represents the action-value function approximated by the i-th Q-network, with $\theta_{i}$ denoting its parameters. The term $y_{j}$ refers to the target Q-value, which is calculated as the minimum value between the two Q-networks, addressing the issue of overestimation. Specifically, the target Q-value $y_{j}$ is computed as follows:

(4) $$y_{j}=r_{j}+\gamma \underset{i=1,2}{\min}Q_{{\theta_{i}}^{\prime }}\left(s_{j+1},\pi_{\emptyset^{\prime }}\left(s_{j+1}\right)+\epsilon \right)$$

Here, $Q_{{\theta_{i}}^{\prime }}$ represents the target Q network, where ${\theta_{i}}^{\prime }$ are the parameters slowly updated from the current Q network parameters $\theta_{i}$. This gradual update process helps stabilize training by providing more consistent target Q-values. The reward $r_{j}$ is obtained after executing action $a_{j}$ in state $s_{j}$, and γ is the discount factor. The target policy $\pi_{\emptyset^{\prime }}$ is used to compute the next action, with ϵ being a small noise added to encourage exploration.

To further enhance stability, TD3 implements delayed policy updates and soft target network updates to further stabilize the learning process. These enhancements ensure more accurate Q-value estimation and robust training, setting TD3 apart from other reinforcement learning algorithms like DDPG. TD3’s robustness in managing continuous action spaces, coupled with its enhancements for stability and efficiency, makes it perform well in crowd navigation.

## 4. Navigation Policy Framework

In this section, we present the design of the navigation policy for the proposed system.

### 4.1. Observation Space

In our proposed method, the observation feature representation, which is used as the input to the TD3 network, is composed of three distinct components, each providing crucial information for effective navigation. First, a 1024-dimensional vector is extracted from the depth image via a CNN. This vector captures spatial features of the environment, helping the robot identify obstacles and free spaces. Second, the depth value of the target object, represented as a scalar, is derived from the depth at the center point of the bounding box detected by YOLO, providing essential target distance information. Lastly, a 128-dimensional vector is produced by a SAN, which processes the historical context of the last 20 actions and the last 20 target positions. This vector captures temporal dependencies and relationships between past actions and target positions, enhancing the robot’s ability to make informed decisions based on prior experiences. By integrating these three components, the TD3 network is equipped with comprehensive spatial and temporal information, allowing it to navigate complex environments and efficiently track the target.

### 4.2. Action Space

The action space is represented by a two-dimensional vector, $a=\left[v,\omega \right]$, where v denotes the linear velocity and ω represents the angular velocity. Actions are selected based on a deterministic policy π, conditioned on the current observation o. Specifically, the action a is sampled from the policy distribution π(a|o). The linear velocity v ranges from 0 to 0.55 m/s, allowing control over the forward speed, while the angular velocity ω represents the angular velocity ranging from −1.5 to 1.5 rad/s, allowing the robot to adjust its heading during navigation.

The limits for the linear and angular velocities are selected based on the hardware specifications of the real robot employed in this study, which ensure that the actions generated by the trained policy in the simulation environment are compatible with the robot’s physical capabilities to address the sim-to-real gap. By matching the velocity limits to the robot’s motor capabilities, reliable movement is achieved while minimizing excessive inertia, thereby ensuring robust and efficient performance during real-world navigation. 

### 4.3. Reward Function

The reward function in our approach is designed to guide the robot toward efficient and safe navigation in dynamic environment. It is defined as follows:

(5) $$R=R_{step}+R_{dt}+R_{cv}+R_{tp}+R_{od}+R_{cr}$$

#### 4.3.1. Step Penalty $R_{step}$

This component encourages the robot to reach the target efficiently by imposing a constant penalty for each time step. It discourages prolonged navigation and oscillatory behavior. The penalty is set at a constant value of

(6) $$R_{step}=-18$$

#### 4.3.2. Distance Reward $R_{dt}$

The distance reward is designed to incentivize the robot to move closer to the target. The reward is proportional to the change in distance between the current position and the target, denoted by $d_{t}$, and the previous distance, $d_{t-1}$. The robot is penalized for moving away from the target:

(7) $$R_{dt}=-150\;\cdot \left(d_{t}-d_{t-1}\right)$$

#### 4.3.3. Camera View Reward $R_{\mathrm{cv}}$

This reward component encourages the robot to keep the target within the camera’s view. The robot is rewarded when the target is visible and penalized when it is lost. The reward is based on whether the target’s bounding box is detected.

(8) $$R_{cv}=\left\{\begin{array}{c} \;\;\;10,\;\;\;\;\;if\;the\;\;target\;\;is\;\;detected\\ -10,\;\;\;\;\;otherwise \end{array}\right.$$

#### 4.3.4. Target Position Reward $R_{tp}$

This reward component encourages the robot to center the target in its view. The closer the target to the center of the camera’s view, the higher the reward. The position of the target within the view is denoted as $P_{target}$.

(9) $$\;R_{tp}=\left\{\begin{array}{c} 15\cdot \left(1-\left|P_{target}\right|\right),\;\;\;\;\;if-1\leq P_{target}\leq 1\\ \;\;\;-15,\;\;\;\;\;otherwise \end{array}\right.$$

#### 4.3.5. Obstacle Distance Penalty $R_{od}$

This term penalizes the robot for moving too close to obstacles. The penalty is proportional to the change in distance between the robot and the nearest obstacle, where $d_{o_{t}}$ and $d_{o_{t-1}}$ represent the current and previous distance, respectively:

(10) $$R_{od}=3000\cdot \left(d_{o_{t}}-d_{o_{t-1}}\right)$$

#### 4.3.6. Collision Risk Penalty $R_{cr}$

**Figure 5:**
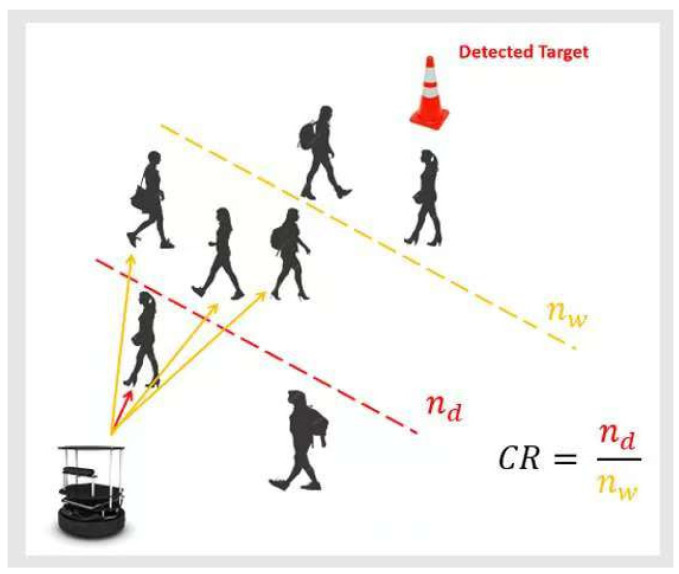
The collision risk penalty based on the ratio of the number of “dangerous-level” depth pixels to the number of “warning-level” depth pixels.

A collision risk penalty is introduced to penalize the robot for operating in high-risk areas. As illustrated in Figure 5, it is calculated as the ratio of the number of “dangerous-level” depth pixels $n_{d}$, where their depth is below a danger threshold, to the number of “warning-level” depth pixels $n_{w}$, where their depth is below a warning threshold:

(11) $$CR=\frac{n_{d}}{n_{w}}\;$$

The corresponding penalty is as follows:

(12) $$R_{cr}=-CR\cdot 180$$

The collision risk penalty encourages the robot to avoid highly crowded areas and choose a less crowded area for navigation.

#### 4.3.7. Design Principles of Reward Function

The reward function is designed based on two fundamental principles: (1) the ultimate objective is to reach the target location, and (2) the robot must avoid obstacles along the way. These principles synergize to guide the robot’s behavior, ensuring efficient movement toward the target while minimizing collisions and navigational inefficiencies.

In accordance with the first principle, the robot is incentivized to move toward the target through the “distance reward”, $R_{dt}$, which is assigned a substantial negative weight of −150. This strong penalty discourages deviations and promotes consistent progress toward the target. While keeping the target near the center of the robot’s FOV is beneficial for navigation, it is considered less critical. Therefore, the “target position reward”, $R_{tp}$, is assigned a relatively lower gain of ±15. Additionally, to account for scenarios where temporarily losing sight of the target may facilitate better obstacle avoidance, the “camera view reward”, $R_{cv}$, is assigned a low gain of ±10. This balance ensures that the robot prioritizes progress toward the target while remaining flexible in its navigation strategy.

In alignment with the second principle, the reward function incorporates components that penalize risky proximity to obstacles. The “collision risk penalty”, $R_{cr}$, acts as a preventive measure, signaling danger and discouraging the robot from navigating through crowded areas. This is achieved with a strong penalty weight of −180. Furthermore, the “obstacle distance penalty”, $R_{od}$, penalizes the robot which is extremely near an obstacle. This component carries an extremely high penalty of 3000 to strongly encourage rapid evasive actions to prevent imminent collisions.

## 5. Model Training

This section outlines the training process for the proposed model, which integrates both CNN and SAN modules within a DRL framework.

### 5.1. Network Training

By combining the spatial information extracted by the CNN with the temporal dependencies captured by the SAN, the model addresses the limitations posed by the robot’s constrained FOV. This fusion enables more informed and anticipatory decision-making, particularly in dynamic and crowded environments.

**Figure 6:**
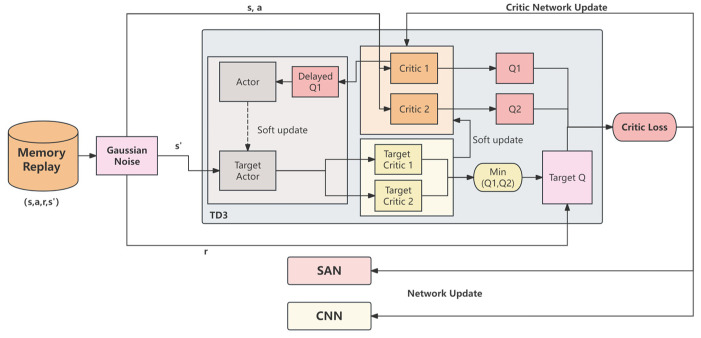
Enhanced TD3 framework for visual target-driven robot crowd navigation with simultaneous CNN and SAN updates using critic loss.

Figure 6 illustrates the overall framework of our proposed enhanced TD3 algorithm, where the critic loss of TD3 network is used not only to update the traditional critic network but also to simultaneously update the CNN and SAN. Specifically, the state–action–reward–next state $\left(s,\;a,\;r,\;s^{\prime }\right)$ data in the experience replay buffer are first processed with Gaussian noise to generate a noisy next state $s^{\prime }$. At this point, the TD3 algorithm uses two critic networks (Q1 and Q2) to compute the Q-values, and the target Q-value $y_{j}$ is generated by taking the minimum of the two target critic networks (Target Critic 1 and Target Critic 2), which is then used to calculate critic loss.

In standard TD3, the critic loss is solely used to update the parameters of the critic network itself. However, in our design, the critic loss is further utilized to update the parameters of the CNN and SAN, enabling them to learn features that help reduce the Q-value prediction error. The parameters of the CNN and SAN are updated simultaneously by minimizing this loss function. The gradient descent update rules are given by

(13) $$\theta_{CNN}\;\leftarrow \;\theta_{CNN}-\alpha \nabla_{\theta_{CNN}}L\left(\theta_{1},\theta_{2}\right)\;$$

(14) $$\theta_{SAN}\;\leftarrow \;\theta_{SAN}-\alpha \nabla_{\theta_{SAN}}L\left(\theta_{1},\theta_{2}\right)\;$$

where α is the learning rate, and $\nabla_{\theta_{CNN}}L$ and $\nabla_{\theta_{SAN}}L$ are the gradients of the loss function with respect to the CNN and SAN parameters, respectively. Specifically, the CNN is primarily responsible for extracting visual information from RGB-D images, while the SAN is used to extract temporal features from historical target positions and action sequences. By aligning the CNN and SAN with the target of the critic loss, they can collectively learn state and action features that are beneficial for Q-value prediction, thereby enhancing the accuracy of the Critic network’s Q-value estimation.

Moreover, this joint optimization method ensures consistency between the feature extraction networks and the policy network, reducing the issue of feature mismatch among networks and improving the stability and overall performance of the training process.

We provide the pseudocode, outlined in Algorithm 1, which details the comprehensive steps and procedures employed during the model training process. The pseudocode includes important aspects such as algorithm initialization, the training process, and parameter updates to facilitate the reader’s understanding of the algorithm’s implementation.
**Algorithm 1: Combined Training of TD3 with CNN and SAN**1Initialize critic networks $Q_{i}\;\left(i=1,2\right)$, and actor network $\pi_{\emptyset }$ with parameters $\theta_{i}\;\left(i=1,2\right)$ and ∅
2Initialize target networks ${\theta_{i}}^{\prime }\;\leftarrow \;\theta_{i}\left(i=1,2\right)$, $\emptyset^{\prime }\leftarrow \emptyset$
3Initialize replay buffer B
4while t<train_steps do5   s←env.step()6
    for t=0…T−1 do7$\;\;\;\;a\;\approx \;\pi_{\emptyset }\left(s\right)+\epsilon$8$\;\;\;\;s^{\prime }\;,\;r\;\leftarrow \;env.step\left(a\right)$9     store $\left(s,a,r,s^{\prime }\right)$ into B
10     if learning_mode then11       Sample mini-batch $\left(s,a,r,s^{\prime }\right)$ from B
12       Next action $a^{\prime }\leftarrow \;\pi_{\emptyset^{\prime }}\left(s^{\prime }\right)+\epsilon^{\prime }$
13$\;\;\;\;\;\;\;y\;\leftarrow r+\gamma \underset{i=1,2}{\min}Q_{{\theta_{i}}^{\prime }}\left(s^{\prime },a^{\prime }\right)$14$\;\;\;\;\;\;\;critic\;loss\;L_{\theta_{i}}\leftarrow \frac{1}{N}\sum {\left(Q_{\theta_{i}}\left(s,a\right)-y\right)}^{2}$15         Update critics $\theta_{i}\;\leftarrow \;L_{\theta_{i}}$
16         Update CNN $\theta_{CNN}\;\leftarrow \theta_{CNN}-\alpha \nabla_{\theta_{CNN}}L_{\theta_{i}}$
17         Update SAN $\theta_{SAN}\;\leftarrow \;\theta_{SAN}-\alpha \nabla_{\theta_{SAN}}L_{\theta_{i}}$
18         if t mod d then19            Update ∅ by deterministic policy gradient:20$\;\;\;\;\;\;\;\;\;\;\;\;\;\;\;\nabla_{\emptyset }J\left(\emptyset \right)=\frac{1}{N}\sum \nabla_{a}Q_{\theta_{1}}\left(s,a\right){|}_{a=\pi_{\emptyset }\left(s\right)}\nabla_{\emptyset }\pi_{\emptyset }\left(s\right)$21            Update target networks:22$\;\;\;\;\;\;\;\;\;\;\theta_{i}\;\leftarrow \;\tau \theta_{i}+\left(1-\tau \right){\theta_{i}}^{\prime }$23$\;\;\;\;\;\;\;\;\;\;\emptyset^{\prime }\;\leftarrow \;\tau \emptyset +\left(1-\tau \right)\emptyset^{\prime }$24         end if25     end if26    end for27end while

In TD3, the critic network plays a crucial role in estimating Q-values by minimizing the critic loss, which measures the error between predicted and target Q-values. This process ensures accurate value functions and stable convergence. The CNN and SAN are integral components of the framework, with the former extracting spatial features from RGB-D images and the latter capturing temporal dependencies from historical data. However, when optimized independently, these networks face limitations, as their feature extraction processes are not directly aligned with the reinforcement learning objective of minimizing Q-value prediction errors. This misalignment can lead to feature mismatch, reducing the effectiveness of the overall system.

To address this, we leverage the critic loss to simultaneously update the CNN and SAN, aligning their training objectives with the reinforcement learning goal. This joint optimization provides several key benefits:

- Direct Alignment with RL Objectives: The CNN and SAN no longer optimize features independently but instead learn representations that directly minimize Q-value prediction errors, improving overall efficiency.
- Reduced Feature Mismatch: By aligning feature extraction with the critic’s loss function, the features learned by the CNN and SAN are more consistent and better suited to support the critic network.
- Improved Stability and Performance: The unified training process enhances the stability of the learning process, accelerates convergence, and improves policy performance by ensuring that spatial and temporal features are optimized to complement each other.
- Enhanced Feature Representation: The CNN and SAN learn richer and more task-relevant features, enabling the critic network to model complex environments effectively.
- Avoiding Optimization Conflicts: Jointly training the CNN and SAN avoids potential conflicts or redundancies that may arise when these networks are optimized independently.

This integrated design allows the model to utilize spatial and temporal information more efficiently, compensating for limited FOV and enabling robust decision-making in dynamic and crowded environments.

### 5.2. Training Environment and Procedure

**Figure 7:**
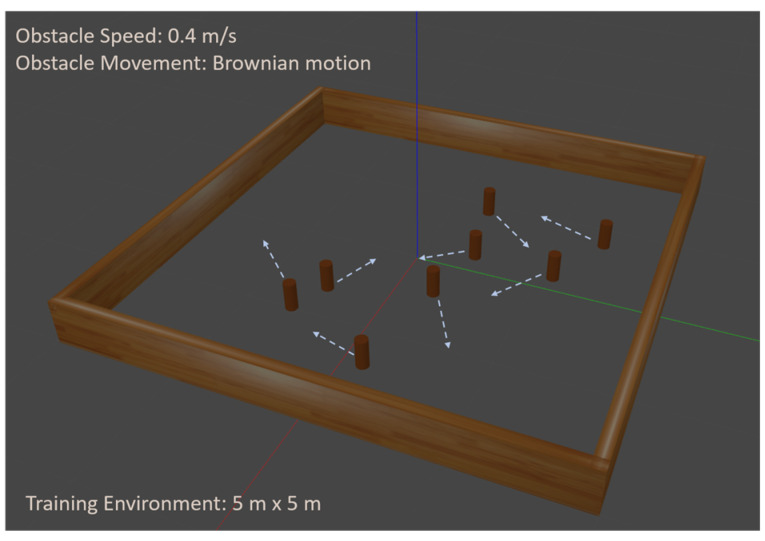
Simulation environment for training and testing. The arrows indicate obstacle movements.

The model training was conducted using an NVIDIA RTX 3090 GPU running Robot Operating System (ROS) Noetic distribution and Gazebo simulator. The simulated environment, as shown in Figure 7, comprises a 5 m × 5 m square area populated with 12 dynamic obstacles.

These obstacles are cylindrical in shape with a height of 0.24 m and a radius of 0.05 m, exhibiting random Brownian motion with a maximum linear speed of 0.4 m/s. The autonomous robot, a TurtleBot Burger equipped with an Intel RealSense D435 depth camera, was tasked with navigating this environment. The robot’s mobility constraints were set with a maximum linear velocity of 0.55 m/s and a maximum angular velocity of 0.15 rad/s. The depth camera provides both depth and visual input, which are subsequently processed to extract crucial environment information for navigation.

Over the course of training, the robot underwent 120,000 episodes, totaling approximately 185 h. Each episode initiated with the robot positioned randomly within the environment, aiming to reach a predefined target location while avoiding collisions with the moving obstacles. The primary training objective focused on maximizing the cumulative reward, designed to incentivize efficient and safe target pursuit. Throughout the training phase, the robot’s policy was continually refined via the TD3 algorithm. This enhancement was supported by a SAN, which leveraged both spatial and temporal information from historical observations and target.

This training setup enabled the robot to learn navigation strategies within a crowded and dynamically changing environment, achieving real-time decision-making under limited FOV.

## 6. Results

We tested our approach in a simulated environment with different obstacle movement patterns and conducted evaluations of our method across three distinct scenarios to assess its performance relative to existing DRL-based approaches. Our comparison was made with (1) map-based DRL approaches in static environments, (2) vision-based DRL approaches in static environments, and (3) TD3-based approach in dynamic environments.

### 6.1. Trajectory Visualization in Dynamic Environment

**Figure 8:**
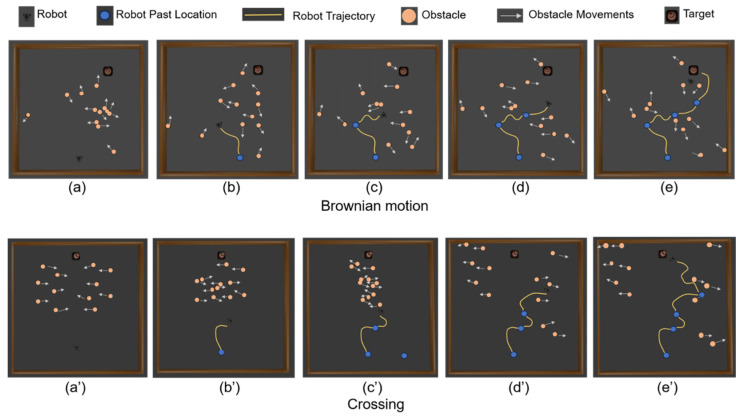
Trajectories of the robot navigating a 5 m × 5 m simulated space with eight moving cylindrical obstacles. (**a**–**e**) illustrate the robot’s trajectory in an environment where the obstacles move in a Brownian pattern, while (**a’**–**e’**) illustrate the robot’s trajectory in an environment where the obstacles move in a crossing pattern.

To evaluate the robot’s navigation performance in dynamic environments, we visualized its trajectory in a simulated space. Figure 8 illustrates a typical trajectory of a robot navigating a 5 m × 5 m simulated space populated with eight cylindrical obstacles, each measuring 0.24 m in height and 0.05 m in radius. These obstacles are programmed to move in patterns of Brownian motion and crossing motion, with a maximum velocity of 0.4 m/s. The robot leverages its RGB-D camera to capture frames representing its state, comprising both RGB and depth data, at successive time intervals as it progressively navigates toward the target in a dynamic environment. The variations in the trajectory confirm the robot’s real-time adaptability to the movements of the obstacles, demonstrating the effectiveness and flexibility of the self-attention enhanced DRL approach in complex navigation tasks.

### 6.2. Comparison with Map-Based DRL Approaches in Static Environment

Our experimental setup included a comparative evaluation against map-based DRL approaches. Our method achieved a success rate of 0.97 based on 100 test episodes in a mapless, target-driven simulation environment utilizing a single RGB-D camera. This result outperforms other DRL-based methods in similar static environments, as summarized in Table 1.

It is important to highlight that these baseline methods depend on prebuilt maps and a predefined fixed target location, substantially simplifying the navigation task compared to our mapless, visual target-driven setup. In our trials, we employed a 5 m × 5 m indoor environment with four randomly placed static cylindrical objects and a randomly located target object. Despite the challenging conditions, our method demonstrated superior navigation capabilities, achieving the highest success rate, and illustrating its adaptability and efficacy in unstructured, mapless environments, which is a crucial advantage for real-world applications where prebuilt maps are unfeasible.

### 6.3. Comparision with Mapless Vision-Based DRL Approaches in Static Environment

In further evaluations, our method was contrasted against other vision-based DRL strategies in indoor environments, as detailed in Table 2. Our approach recorded a success rate of 0.87 based on 100 test episodes in the simulation environment with eight obstacles in Brownian motion, utilizing a single RGB-D sensor for perception. In contrast, the method developed by Wu et al. [26], tested in a static indoor bathroom using the AI2-THOR framework, achieved a success rate of 0.627. The PPAC+LSTM method [25] reported a perfect success rate of 1.0 in a simulated static indoor bathroom scenario, without any moving obstacle. The DS-DSAC with the PredRNN++ [36] method reported a 0.86 success rate in large indoor offices. Similarly, the Goal-Directed method proposed by Zhou et al. [37] achieved a 0.82 success rate to navigate to specific rooms from a corridor.

Our test environment, a 5 m × 5 m simulated space populated with eight cylindrical obstacles each 0.24 m in height and 0.05 m in radius moving at a maximum velocity of 0.4 m/s in a Brownian motion, represents a more complex and realistic challenge compared to the static conditions of the baseline methods. This demonstrates the robustness and adaptability of our method, offering significant benefits for practical robotic navigation, especially when employing a vision sensor with limited FOV.

### 6.4. Comparision with Mapless Vision-Based DRL Approaches in Dynamic Environment

In further tests, we compared our method with a baseline TD3 algorithm [13] under varying obstacle densities (4, 8, and 12 obstacles) in Brownian motion. Using a single RGB-D camera, our approach achieved success rates of 0.93, 0.87, and 0.57, respectively, based on 100 test episodes per scenarios, outperforming the baseline TD3 method running in our simulation environment, as summarized in Table 3.

We extensively tested the open-source program of [13] in the same simulated environment and recorded its average performance in Table 3. This baseline method, while achieving similar performance in low-density environments, showed significant performance declines in higher obstacle densities. This performance indicates that our approach not only maintains competitive success rates but also facilitates faster and more efficient navigation, validating its effectiveness in visual target-driven robot navigation tasks under varying dynamic conditions.

### 6.5. Study on Limited FOV

To investigate the impact of FOV on the robot’s navigation performance, we designed an experiment by progressively cropping the original RGB and depth images to simulate varying FOVs. The original resolutions of the RGB and depth data are 1920 × 1080 pixels and 1280 × 720 pixels, respectively. The images were cropped at their borders in 10% increments for both RGB and depth data, as detailed in Table 4. The experiment was conducted in a simulation environment with eight dynamic obstacles moving in Brownian motion. For each scenario, we evaluated the navigation policy based on success rate and average time, based on 100 test episodes per scenario.

### 6.6. Ablation Study

**Figure 9:**
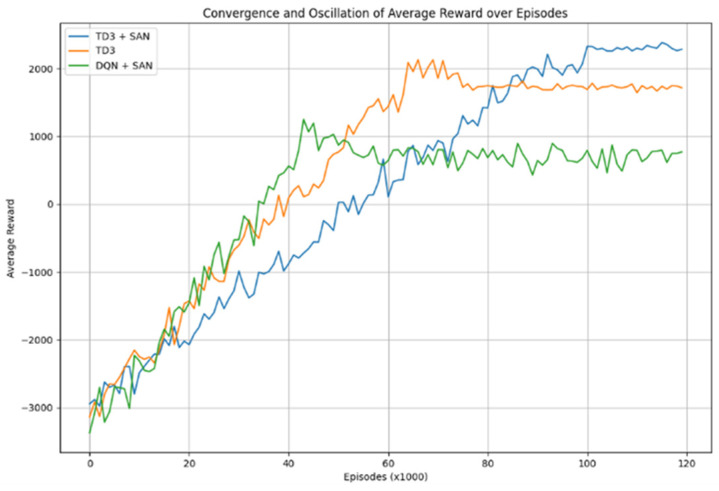
Learning curves in the ablation study.

To determine the efficacy of various reinforcement learning configurations, we conducted an ablation study comparing three configurations with respect to their learning convergence and reward maximization. The result is shown in Figure 9.

#### 6.6.1. TD3 + SAN

This configuration exhibited stable convergence throughout the training period. Initially, the reward values progressively increased from −3000, approaching a level of approximately 2000 within the first 90,000 episodes. After this phase, the reward plateaued around 2300, indicative of high performance and long-term stability.

#### 6.6.2. Standalone TD3

Standalone TD3: In contrast, the standalone TD3 algorithm demonstrated faster convergence compared to the TD3 + SAN, with rewards rising from the initial values and stabilizing near 1900. Despite some fluctuations observed between episodes 65,000 and 75,000, the final reward consistently hovered around 1900, suggesting quicker convergence but at a lower reward threshold compared to the TD3 + SAN.

#### 6.6.3. DQN + SAN

The DQN + SAN configuration showed the least favorable outcomes among the three tested configuration. Starting with an initial reward of −3400, it exhibited modest improvement during the first 45,000 episodes before stabilizing at a final reward of approximately 920. This configuration suffered from the highest degree of oscillation and demonstrated substantial instability, marking it as the least effective in this task.

It can be concluded from the ablation study that the TD3 + SAN configuration outperformed the others by achieving the highest final rewards and exhibiting significant training stability, albeit with a slower convergence rate. Conversely, the TD3 algorithm, though converging faster, failed to achieve the higher reward levels of the TD3 + SAN. DQN + SAN displayed considerable instability and underperformance, reinforcing its unsuitability for robust task execution.

### 6.7. Experiment Using a Real Robot

**Figure 10:**
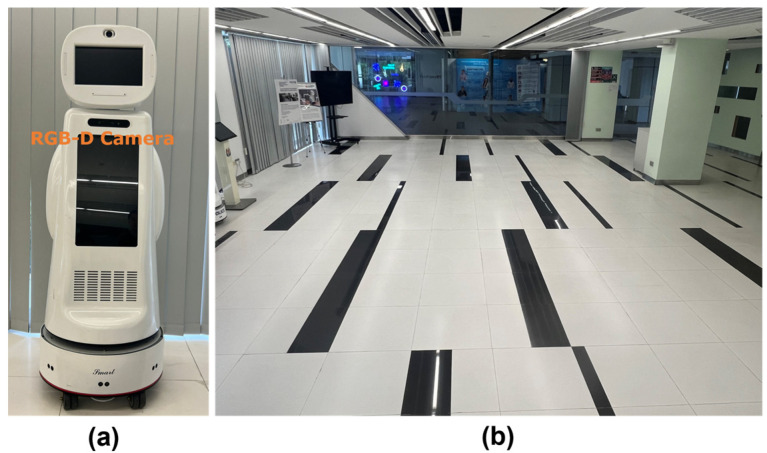
Real robot experiment. (**a**) Real robot with RGBD camera. (**b**) Experiment environment.

To validate the proposed visual target-driven navigation approach, real-world experiments were conducted using a mobile robot, as shown in Figure 10. The robot was equipped with an RGB-D camera, which provided a 70-degree field of view, and operated under ROS Noetic. The experiments were carried out in a 9.5 m × 7 m indoor environment, with the robot and the target positioned at opposite ends of the area. To simulate a dynamic environment, five pedestrians were included, moving at typical walking speeds to represent moving obstacles. The safety cone served as the visual target for the robot.

The failure condition was defined as the robot coming within 6 cm of a human or a wall, as detected by an ultrasonic sensor, to ensure safety. Additionally, if the robot failed to reach the target within 30 s, the trial was considered a failure. The success condition was defined as the robot coming within 15 cm of the target, as detected by the RGB-D camera.

**Figure 11:**
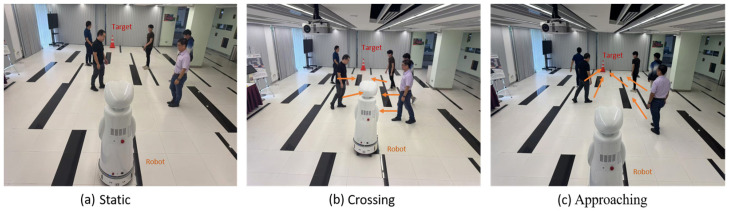
Real robot operating in three scenarios. The arrows in (**b**) and (**c**) indicate human movements.

Three distinct scenarios were tested to evaluate the performance of the trained policy: (a) pedestrians standing randomly along the pathway from the robot to the target without movement; (b) pedestrians positioned on either side of the pathway, with some crossing the pathway; and (c) pedestrians positioned on either side of the pathway, with some walking toward the target. The experimental setup is shown in Figure 11. Each scenario was tested 20 times, and the success rate and average passing time for each trial are summarized in Table 5.

## 7. Discussion

### 7.1. Benefits of TD3 + SAN

In Figure 8, the incorporation of the SAN led to a significant improvement in performance, particularly in the TD3 + SAN configuration. This improvement can be attributed to the SAN’s ability to enable the robot to dynamically focus on relevant information and recover lost targets when the target temporarily leaves its FOV. By leveraging past observations and capturing global contextual relationships, the SAN helps the robot infer the target’s approximate position and resume tracking effectively. Specifically, the SAN offers two key advantages:

- Target Recovery and Tracking: Through its relational reasoning capability, the SAN correlates spatial and temporal patterns in previous observations, allowing the robot to infer the target’s location even in scenarios with occlusions or limited visibility.
- Dynamic Focus on Critical Regions: The mechanism of the SAN enables the robot to dynamically assign importance weights to different regions in the observation space. This allows the robot to prioritize target- and obstacle-related information, enhancing decision-making under uncertainty.

Compared to traditional approaches, the SAN improves the robot’s robustness to partial observability, reduces instability, and ensures smoother navigation trajectories. These advantages are evident in the TD3 + SAN results, where the robot achieved higher rewards and greater long-term stability than TD3 alone.

### 7.2. Overcoming the Challenge of Limited FOV

In visual target-driven crowd navigation, the limited FOV often causes the target to be lost, hindering navigation performance. Our method integrates the SAN, which leverages information from past frames to help the robot recover and track the target effectively. Additionally, we utilize the TD3 algorithm, which is particularly well suited for handling continuous action spaces, to enable the robot to efficiently navigate and reach the target. TD3’s Actor-Critic mechanism, along with its key features of target value smoothing and delayed updates, ensures stable learning, allowing the robot to adapt to dynamic crowd environments even with a limited FOV. Moreover, our reward function design of camera view reward $R_{cv}$ and target position reward $R_{tp}$ effectively encourages the robot to keep the target within its view.

The results of the TD3 + SAN demonstrate that the combination of the SAN and TD3 enhances the robot’s robustness and navigation performance under limited FOV conditions, validating the effectiveness of our approach.

### 7.3. Collaborate Training of CNN, SAN, and TD3 for Better Performance

The experimental results are shown in Figure 9. The TD3 + SAN demonstrates significant advantages in dynamic and crowded environments with limited field of view. Compared to traditional TD3 and DQN + SAN, the TD3 + SAN converges to a higher average reward more quickly, exhibits better stability, and achieves significantly better final reward values. This indicates that the collaborative optimization of the CNN and SAN effectively utilizes both visual and temporal features, compensating for the perception limitations due to the restricted field of view, while enhancing decision-making robustness and performance in complex interaction scenarios.

### 7.4. Performance in Real Deployment

In the simulation environment, experiments were conducted on two scenarios: Brownian motion and crossing. The success rate for crossing was lower than that for Brownian motion, primarily because the robot is often surrounded by obstacles during crossing. In real-world deployment, simulating Brownian motion is not feasible. Instead, experiments were conducted on crossing and approaching scenarios. Unlike in the simulation, human movement speeds are variable, as individuals may move faster or slower, further reducing the robot’s success rate. In the approaching scenario, obstacles frequently formed a “wall” around the robot, causing it to lose track of the target. The robot often failed to recover the target before timing out, which significantly impacted its success rate.

## 8. Conclusions

In this study, we proposed a new visual target-driven navigation strategy for robots operating in crowded and dynamic environments with a limited FOV. Our approach utilizes the TD3 algorithm and integrates the SAN, enabling effective navigation without the need for pre-mapped environments.

Our contributions offer meaningful advancements in the field of autonomous robotic navigation. Firstly, we successfully reduced hardware and computational requirements by employing a single RGB-D camera, thereby simplifying the system’s architecture, and lowering the overall cost and complexity. Secondly, the incorporation of a SAN enabled our system to maintain awareness of the navigation target even with a limited sensory field, enhancing the robot’s ability to relocate and track dynamic targets efficiently. Finally, the robust performance of our approach was demonstrated through extensive simulation and real robot experiments, which showed superior navigation capabilities in terms of both obstacle avoidance and target pursuit in complex environments.

The experimental results validated that our model outperforms traditional DRL-based methods in dynamic environments, achieving higher success rates and shorter average target-reaching times. Notably, our method exhibited greater adaptability to changes within the environment, proving particularly effective in real-world scenarios where unpredictability and the presence of dynamic obstacles are common.

In future work, we plan to systematically investigate the impact of hardware configurations on the performance of the proposed navigation policy across various scenarios. For example, a lower camera resolution may reduce the system’s accuracy in detecting targets and obstacles, thereby negatively affecting navigation performance and success rates. Future research could focus on developing adaptive policies that dynamically adjust to varying hardware constraints or explore hardware augmentation strategies to enhance the robustness and reliability of navigation in complex and dynamic environments.

## Figures and Tables

**Table 1:** Performance comparison with map-based DRL approaches.

Method	Success Rate	Sensor	Env	Target
Ours	0.97	RGB-D	Mapless	Randomly located
DQN-SLAM [20]	0.94	LiDAR	Map-based	Predefined
PPO-SPD [21]	0.996	RGB-D + LiDAR	Map-based	Predefined
LSTM-LMC [33]	0.92	RGB-D	Map-based	Predefined
A1-RD [34]	0.65	LiDAR	Map-based	Predefined
Method by Yang et al. [35]	0.94	LiDAR	Map-based	Predefined

**Table 2:** Performance comparison with vision-based DRL approaches in mapless scenarios.

Method	Success Rate	Environment	Sensor
Ours	0.87	With 8 moving obstacles	RGB-D
Method by Wu et al. [26]	0.627	Static environment	RGB
PPAC+LSTM [25]	1.0	Static environment	RGB-D
DS-DSAC with PredRNN++ [36]	0.86	Static environment	RGB-D
Goal-Directed method by Zhou et al. [37]	0.82	Static environment	RGB

**Table 3:** Performance comparison with existing TD3-based approach in mapless scenarios.

Method	Success Rate			Average Time (s)			Sensor	Target
	4 moving obstacles	8 moving obstacles	12 moving obstacles	4 moving obstacles	8 moving obstacles	12 moving obstacles		
Ours	0.93	0.87	0.57	13.78	15.96	18.87	RGB-D	Randomly located
TD3 with collision probability [13]	0.93	0.82	0.49	22.98	27.13	37.13	LiDAR	Predefined

**Table 4:** Navigation performance under different FOVs.

FOV	Success Rate	Average Time
Original	0.87	15.96 s
10% of FOV cropped	0.81	19.71 s
20% of FOV cropped	0.66	26.27 s
30% of FOV cropped	0.49	33.81 s

**Table 5:** Results of the real robot experiment.

Motion Type	Success Rate	Average Time
Static	18/20	12.37 s
Crossing	15/20	19.32 s
Approaching	11/20	23.47 s

## Data Availability

The original contributions presented in the study are included in the article. Further inquiries can be directed to the corresponding author.
